# Case management in oncology rehabilitation (CAMON): The effect of case management on the quality of life in patients with cancer after one year of ambulant rehabilitation. A study protocol for a randomized controlled clinical trial in oncology rehabilitation

**DOI:** 10.1186/1745-6215-12-103

**Published:** 2011-04-28

**Authors:** Irene Bachmann-Mettler, Claudia Steurer-Stey, Oliver Senn, Mathyas Wang, Katarina Bardheci, Thomas Rosemann

**Affiliations:** 1 Institute of General Practice and Health Services Research, University of Zurich, Zurich, Switzerland

## Abstract

**Background:**

Cancer diseases and their therapies have negative effects on the quality of life. The aim of this study is to assess the effectiveness of case management in a sample of oncological outpatients with the intent of rehabilitation after cancer treatment. Case management wants to support the complex information needs of the patients in addition to the segmented structure of the health care system. Emphasis is put on support for self-management in order to enhance health - conscious behaviour, learning to deal with the burden of the illness and providing the opportunity for regular contacts with care providers. We present a study protocol to investigate the efficacy of a case management in patients following oncology rehabilitation after cancer treatment.

**Methods:**

The trial is a multicentre, two-arm randomised controlled study. Patients are randomised parallel in either 'usual care' plus case management or 'usual care' alone. Patients with all types of cancer can be included in the study, if they have completed the therapy with chemo- and/or radiotherapy/surgery with curative intention and are expected to have a survival time >1 year. To determine the health-related quality of life the general questionnaire FACT G is used. The direct correlation between self-management and perceived self-efficacy is measured with the Jerusalem & Schwarzer questionnaire. Patients satisfaction with the care received is measured using the Patient Assessment of Chronic Illness Care 5 As (PACIC-5A). Data are collected at the beginning of the trial and after 3, 6 and 12 months. The power analysis revealed a sample size of 102 patients. The recruitment of the centres began in 2009. The inclusion of patients began in May 2010.

**Discussion:**

Case management has proved to be effective regarding quality of life of patients with chronic diseases. When it comes to oncology, case management is mainly used in cancer treatment, but it is not yet common in the rehabilitation of cancer patients. Case management in oncology rehabilitation is not well-established in Switzerland. A major challenge of the study will therefore probably be the recruitment of the patients due to the physicians' and patients' scarcely existing awareness of this issue.

**Trial registration:**

ISRCTN41474586

## Background

Cancer diseases and their therapies have short- and long-term negative effects on the quality of life. Thus, it is important to offer patients targeted rehabilitation and integrated care [1]. Analyses of 'survivors' after a five-year survival time revealed various psychosocial problems with impact on the patient's quality of life and career depending on the illness and care received [2,3]. Since cancer diseases develop into chronic conditions, patients not only expect physical rehabilitation, but also a broad range of services offered to develop skills which can enable them to cope with the long term consequences of cancer diseases [4,5]. For this reason provision of individual- and group-oriented rehabilitation programs satisfies the patients' demands for continuity in care and for encouragement to develop self-management skills as described in the Chronic Care Model of the World Health Organization (WHO) [6,7]. This is in accordance with Bergeson et al [8], who call for an efficient care network for the chronically ill to contain the following: 1. Access to and continuity in care, 2. Close involvement of patients in the provision of their own care, 3. Supportive measures for self-help or self-management, respectively, 4. Coordination of care between individual institutions and service providers.

Ambulant rehabilitation following cancer therapy is not well established in Switzerland. Despite the fact that psychosocial counselling, psycho-oncological therapy and opportunities to increase physical fitness are widely offered, the needs of the patient cannot be met. According to patient surveys the following problem areas were defined: inadequate information on side-effects and on consequences of the acute treatment, lack of process in communication, problematic transition from acute care to aftercare and rehabilitation, serious economical consequences and insufficient preparation for return to work. Patients value additional information and support on the following topics: information on cancer diseases, conduct in case of therapy and handling thereof in daily life, an overview of offers of support and counselling, advice on and guidance in matters related to pain, fatigue, dietary change, hair loss, and complementary or alternative medical therapies [9-12]. Similarly, patients expect to be offered physical movement programs and counselling on psychological strain and the resumption of daily chores/work, financial matters and worries, as well as counselling for relatives. Patients in rehabilitation have the following objectives: physical fitness, dietary changes, weight reduction and capacity to deal with psychological stress. They have the following expectations of their doctors: holistic understanding of rehabilitation with coordinated services offered targeting the various aspects of quality of life, information on rehabilitation services offered, reintegration into everyday and professional life [9-12]. Dissatisfaction of the patient does not arise due to a lack of rehabilitation and advisory services, but rather due to insufficient information on existing offers and failure of doctors to document their needs and consecutively failure to refer them to the appropriate therapeutic and counselling services. After cancer therapy, many patients do not have the energy to get themselves the necessary information they need and to claim for support. Case management, where a case manager (rehabilitation coach) provides coordination and exchange of relevant information between doctors and other care providers, is therefore one way to tackle these problems actively.

## Case Management

Case management (CM) used in the care of the chronically ill, is described in several ways; targets and interventions range from coordinating multidisciplinary therapies with adequate provision of the relevant information to patients and therapist to the assessment of patients' needs and thus the appropriate interventions. Case management can function as a communication centre for all parties involved, but can also serve as a main contact person (primary nurse) [13-16]. Consequently, CM wants to support the complex information needs of the mostly chronically ill patients taking into account the segmented structure of the health care system [13]. Hence, emphasis is put on support for and activation of self-management in order to enhance health- conscious behaviour, learning to deal with the burden of the illness and providing the opportunity for regular contacts with care providers. This approach is part of the Chronic Care model of the WHO. CM is a collaborative process adhering to a system, that tries to compile patient needs and resources, goals of rehabilitation, planning and execution of interventions and evaluations in collaboration with the patient, and, if necessary with their relatives. Considering oncology, CM is successfully applied in the follow-up after therapies and can help to improve the recording of cancer treatments and its symptoms [17,18]. However, a systematic review comprising seven trials found no concluding evidence regarding the effectiveness of case management due to differences in interventions and trial endpoints [19].

### Fostering self-management

It is assumed that the fostering of self-management helps patients to manage the rehabilitation phase more easily and to develop skills to improve their health status. Adequate self-management requires a lot from the chronically ill patient. Suffering from cancer often causes mental stress, fear, uncertainty and insecurity for one's life and future. Effective self-management programs aim to support patients in developing strategies to positively improve their health status. The encouragement of self-efficacy is a relevant concept, that means to promote an optimistic outlook as well as faith in the ability to handle new or difficult situations by means of one's own competence [20]. Many patients need additional counselling and coaching to develop the skills needed to deal with the sequels of illness and medication, with stress and other emotional strain; dietary changes; the promotion of physical fitness through exercise; the continuation or adjustment of the patient's role in the family, and in their professional and daily life. The following five elements in self-management programs should be encouraged the most: problem solving; decision making; use of resources; building a relationship between patient and care team; implementation of an action plan [21].

## Methods

### Objectives

The primary goal of the trial is to assess whether the quality of life of cancer patients with usual care plus case management is significantly better compared to usual care alone one year after a first therapy.

#### Secondary outcomes

a) ability to work recorded in days of sick leave

b) self-efficacy

c) planned health care utilisation (general practitioner/oncologist, specialists and hospitalisation), illustrated as number of contacts

d) unplanned consultations (general practitioner/oncologist, specialists and hospitalisation), illustrated as number of contacts that were unplanned

e) Satisfaction with medical care

### Design

The trial is a multicentre, two-arm randomised controlled study. Patients are randomised parallel in either 'usual care' plus case management or 'usual care' alone.

### Participants

It was originally intended to include patients with breast and colon cancer after completion of an adjuvant therapy. Due to difficulties in the recruitment of enough patients, the inclusion criteria, following agreement with the Ethics Committee, were extended. Patients with all types of cancer fulfilling the inclusion criteria can be included. Eleven oncological centres in the Canton of Zurich participate in the recruitment of patients.

### Inclusion criteria for patients

a) Age ≥18 years

b) Completion of a therapy with chemo- and/or radiotherapy/surgery (longer term hormone and antibody therapy are excluded)

c) Therapy with curative intention

d) Patients with an expected survival time >1 year

e) Increased distress score (3-7 on the Distress Thermometer Likert scale)

f) Intention to undertake ambulant rehabilitation

g) Rehabilitation need/prevailing strain

### Exclusion criteria for patients

a) Patients with metastasis and/or cancer in an advanced stage with palliative therapy

b) Patients with an expected survival time ≤1 year

c) Patients with insufficient knowledge of the German language to participate in counseling and evaluations

d) Patients with severe psychiatric diagnoses or apparent great distress requiring medical psychiatric treatment

e) Completion of therapy longer than one month ago

### Exclusion from study

In case of a relapse or progression of the condition with deterioration of the quality of life and therapy, the patient will leave the study. For ethical reasons, case management will be continued if requested by the patient.

### Interventions

#### Usual care

Usual care includes treatment/follow-up by the oncologist and the GP after completion of the first therapy, according to the usual procedure (usually this is done quarterly during the first year after treatment):

a) Follow-up with regard to course of illness

b) Treatment of delayed adverse effects of therapy

c) Rehabilitation, support directly organised by the oncologist/GP

The rehabilitation (i.e. physiotherapy, physical movement/sports, nutritional advice) and the psychosocial support/counselling (i.e. provided by the Swiss Cancer League and/or psycho-oncologists) correspond with the individual needs of the patients and will, as is current practice, be arranged either by initiative of the patient and/or referral by the medical practitioner. There are no restrictions regarding rehabilitation measures and support. A standardisation of 'usual care' to avoid a bias would contradict the individual needs of the patient.

## Case Management

Table 1 shows a summary of the procedure and content of case management (table 1).

**Table 1:** Summary of the procedure and content of case management.

Procedure	Method	Content
1st consultation	Assessment of needs: setting goals/measures according to standardised procedure (CM control system/self-management) Provision of information Ambulant rehabilitation Approach: Motivational counselling Resource-oriented Empathic conduct	Get to know patient, establish relationship, clinical recording of overall situation: symptoms, side effects of therapies, psychosocial situation and distress, impairment of quality of life. If required by patient: clarification on rehabilitation offered, contact person in case of queries and problems

2nd consultation 3rd consultation	Counselling/Instruction Guidance based on targets &measures Approach as above 'Define task/needs'	Develop 'program' in collaboration with patient. Counselling: dealing with symptoms etc. Arrange a therapists, services, e.g. Swiss Cancer League, via GP/oncologist: physiotherapy, nutritional advice, psycho-oncologist, if patient is unable to organise himself. Contact person in case of queries and problems

**Telephone follow-up **at least once per month for approx. 30 minutes during the first 6 months, afterwards reduction if needed. Availability of rehabilitation coaches if required/office hours	Evaluation, possibly new goals, planning Approach as above	Summarising of outcomes, poss. summarise reports of therapists for patient, GP/oncologist. Counselling to encourage self- management. Contact person in case of queries/problems

4th consultation	Evaluation	Concluding interview

The case manager (rehabilitation coach) informs the patient about the appropriate therapeutically and supportive measures and establishes the necessary. The rehabilitation coach offers supplementary patient-orientated advice and instruction and thus encourages self-management. In this way, the belief in self-advocacy through the accomplishment of a more positive mental outlook can be strengthened. The patient is allocated to a rehabilitation coach who will advise and guide the patients during the rehabilitation phase, and, if necessary, during the chronic phase of the illness. The 'motivational interviewing' [22] with the aim to influence behavioural changes by communicating in an accepting and empathic way, serves as basis for communication with patients. The role of the rehabilitation coach is assumed by an experienced, qualified nurse with additional training in oncology care and further training in case management, hired by the Institute of General Practice and Health Services Research, Zurich. For five days and with ongoing weekly training the rehabilitation coaches were prepared and supported in the requirements during a specially designed training program. The equivalent of 120% employment percentage has been made available for the trial to be distributed among four rehabilitation coaches. The program director or research associates/study nurse will not coach the patients.

The intervention with case management lasts for one year. If the patient no longer requires care, the intervention will be concluded earlier.

### Intervention in control group

All patients receive the usual care and counselling after completion of the therapy, as described under the section 'usual care'.

### Measurements

#### Patient characteristics

Demographics include age, gender, education, race/ethnicity, family status, employment status

Medical characteristics include diagnosis and therapies, time since cancer diagnosis, other diagnosis and therapies, supportive therapies.

### Outcome measures

To determine the health-related quality of life (primary outcome), the general questionnaire FACT G version 4 is used. It contains a summary of 27 items divided into four primary focal points relating to quality of life.

To measure secondary outcomes such as ability to work, the days of sick leave will be recorded at work, as well as the help needed with daily chores. The direct correlation between self-management and perceived self-efficacy is measured with the Jerusalem & Schwarzer questionnaire [23]. The satisfaction of patients with the care received is measured using the Patient Assessment of Chronic Illness Care (PACIC 5-A), which has been developed to assess congruency of provided health care to the Chronic Care Model (CCM). Table 2 gives an overview of the measuring tools for the primary and secondary outcomes (table 2). Data is collected at the beginning of the trial, and after 3, 6 and 12 months.

**Table 2:** Overview of measuring tools/measuring time

Outcomes	Measuring tool	Point in time
**Primary Outcome** Quality of life	FACT G	Months 0, 3, 6, 12

**Secondary Outcomes**		Months
Employability	Questionnaire	0, 3, 6, 12
Self - efficacy Unplanned consultations (GP/oncologist, specialist) and hospitalisation Satisfaction with medical care Type, number of rehabilitation measures	Questionnaire self-efficacy expectation by Jerusalem & Schwarzer Number of consultations/rehabilitation- and support measures Questionnaire PACIC 5-A Questionnaire- patient logbook	

### Sample size

The power calculation was based on the following data: mean value in primary outcome (FACT) 22.7, a variation of 3.0 units in the FACT-score is considered clinically relevant and is thus imputed as minimal difference (effect) to be achieved. The standard deviation of FACT is 5.4 according to literature; the level of significance was set at 0.05, also a power at 0.8. The power analysis revealed a sample size of 102 patients. Assuming a drop-out rate of 30% due to the severely ill patient population, 132 patients would need to be enrolled. The calculation of the sample size was done with the aid of Southwest Oncology Group computers.

### Recruitment

**Figure 1:**
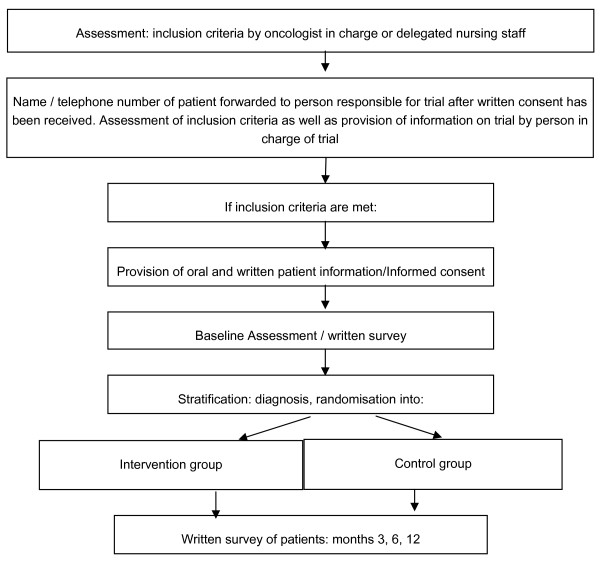
**Workflow of trial**.

Patients who are about to complete or have recently completed cancer therapy (see inclusion criteria), will be informed about the possibility to participate in the trial by their oncologist or the nursing staff. If a patient is interested in participating in the trial, the patient's name and telephone number will be forwarded to the study nurse after written consent has been given. The study nurse will contact the patient, inform him or her orally and in writing about the trial (goals/randomisation/intervention) and clarify the inclusion and exclusion criteria (Figure 1). In order to determine the patient's distress level, the distress thermometer is used [24]. A score above 3 on a Likert scale ranging from 0-10 indicates 'distress'; a score above 7 indicates severe 'distress' and requires medical psychiatric treatment. Severely distressed patients will not be included in the trial, which means that patients with depression can likely be ruled out.

### Randomisation

Treatment allocation will be done by random permutation within blocks with block sizes of 2 and 4. In addition to the block randomization allocation will be stratified according to the type of carcinoma (breast carcinoma, colon carcinoma, Hodgkin and Non Hodgkin lymphoma and one group with "other tumor types") thus allowing for an even distribution of the intervention through the most common expected tumor types with regard to the participating centres various diagnoses. As the size of the participating centres is not necessarily related to the contribution of the participating centres regarding the patient recruitment we will not perform a randomisation stratified by centre. The randomisation is done independently of centres, rehabilitation coaches and the study nurse. The study nurse is responsible for providing patients with detailed information; additionally, they are in charge of the assessment.

The randomisation is performed with STATA statistic program. The relevant information is only accessible to an employee of the Institute of General Practice and Health Services Research who is not involved in the trial. After the inclusion of a patient in the trial and the baseline assessment, the study nurse receives information concerning the allocation of patients to the intervention or control arm by sequentially numbered opaque sealed envelopes. Therefore, the blinding regarding the allocation is guaranteed.

### Statistical analyses

Continuous primary and secondary outcomes will be checked by the Shapiro-Wilk test for normality distribution and data are expressed by means with standard deviations and medians with interquartile range as appropriate. Categorical variables will be expressed as percentages. Primary and secondary outcomes will be compared between groups to investigate the effect of the case management on oncology rehabilitation using parametric or non-parametric tests as appropriate. Categorical outcomes will be analysed by Chi Square tests. To control for any imbalance between groups multiple regression analysis will be used and measures that show any difference in baseline characteristics will be included into the model as potential confounders.

### Timeframe of the study

The recruitment of the centres began in 2009. The inclusion of patients began in May 2010.

### Descriptons of risk

Serious risks or undesired effects of the intervention or the assessment by questionnaires have not been described in the literature. There are no specific risks related to the study.

### Ethical principles

The study is being conducted in accordance with medical professional codex and the Helsinki Declaration as of 1996 as well as Data Security Laws.

Study participation of patients is voluntary and can be cancelled at any time without provision of reasons and without negative consequences for their future medical care.

### Patient informed consent

Prior to study participation patients receive written and oral information about the content and extent of the planned study; for instance about potential benefits for their health and potential risks. In case of acceptance they sign the informed consent form.

In case of study discontinuation all material will be destroyed or the patient will be asked if he/she accepts that the existing material can be used for the study.

### Vote of the ethics committee

The study protocol has been approved by the ethics committee of the Canton of Zurich and received an unrestricted positive vote on 20.5.2010.

### Data security/disclosure of original documents

Patient names and all other confidential information fall under medical confidentiality rules and are treated according to appropriate Federal Data Security Laws. The results of the patient questionnaires are not accessible to the GPs. Questionnaires are directly mailed to the study centre by the patients.

All study related data and documents are stored in a protected central server of the University of Zurich. Only direct members of the internal study team can access the respective files.

Intermediate and final reports are stored in the office of the Institute of General Practice and Health Services Research of the University Hospital of Zurich.

## Discussion

Cancer is a big challenge for the health care system. The number of cancer patients has increased over the last years. With the different improved possibilities of cancer treatment, however, there are an increasing number of patients who survive cancer and are confronted with the sequels of their disease and its treatment. Depending on cancer type and treatment there are several severe consequences which can be overwhelming for patients, so that they often need help. They are confronted with pain, fatigue, distress, diet and rehabilitation needs. Managing all these problems can be a daunting challenge for patients. Concerning the rehabilitation of cancer patients, the lack of systems to recognize rehabilitation needs ranks among the major problems [25]. Screening for rehabilitation needs in the population of oncology outpatients is a bigger challenge than the screening of inpatients [26]. Thus a case manager can provide effective support by indentifying rehabilitation needs and furthermore act as coordinator in the approach of and during rehabilitation measures.

Case management has proved to be effective for example regarding quality of life of patients with chronic diseases namely diabetes, chronic obstructive pulmonary disease and coronary heart disease [27].

When it comes to oncology, case management is mainly used in the follow up of abnormal cancer screening and in cancer treatment, but it is not yet common in the rehabilitation of cancer patients. In a review Robinson-White et al. describe that patient navigation in the population of breast cancer patients seems to be a successful method to encourage women to proceed from breast cancer screening to diagnostic assessment and treatment [28].

Regarding oncology rehabilitation, the effect of case management has not yet been proven. A review including seven papers could not draw any significant conclusions concerning the effectiveness of case management on cancer patients [19]. The authors' explanation for this result is the small number of only seven papers and the significant differences between the case management interventions and effects aimed at. Case management in oncology rehabilitation is not well-established in Switzerland. A major challenge of the study will therefore probably be the recruitment of the patients due to the physicians' and patients' scarcely existing awareness of this issue. The function of people working in the field of case management has various titles such as care coordinator, care/case/disease manager or discharge planner and it includes various skills and a great number of different tasks. A great number of case managers, though, who participated in a cross-sectional descriptive study perceived similar activities and skills as being essential regarding their practice. Considering the evolution of case management, Tahan and Campagna could observe a trend towards professionalization with a growing number of the people concerned having a corresponding degree. With regard to the future development, cost- effectiveness of case management is perceived as one important topic amongst others [29]. Case management seems to be about to find its place in the health care system and confirm its existence. The aim of this study is to assess the effectiveness of case management in a sample of oncological outpatients with the intent of rehabilitation after cancer treatment. The main focus will be on the quality of life. The increasing importance of multidisciplinary care in oncological rehabilitation gives reason for the implementation of the case manager who can assume the role of a coordinator and act as an interface between the different disciplines.

## List of abbreviations used

CCM: Chronic Care Model; CM: Case management; GP: general practitioner; PACIC: Patient Assessment of Chronic Illness Care; WHO: World Health Organization.

## Competing interests

The authors declare that they have no competing interests.

## Authors' contributions

IB conceived of the study and participated in its design and coordination and helped to draft the manuscript. She is responsible of the recruitment of the patients.CS participated in the design of the study and in drafting the manuscript. OS participated in design of the study (randomisation and statistics) and in drafting the manuscript. MW helped to draft the manuscript. KB helped to draft the manuscript. TR conceived of the study and participated in its design and performed the statistical analysis. All authors read and approved the final manuscript.
